# A high-density BAC physical map covering the entire MHC region of addax antelope genome

**DOI:** 10.1186/s12864-019-5790-2

**Published:** 2019-06-11

**Authors:** Chaokun Li, Longxin Chen, Xuefeng Liu, Xiaoqian Shi, Yu Guo, Rui Huang, Fangyuan Nie, Changming Zheng, Chenglin Zhang, Runlin Z. Ma

**Affiliations:** 1 State Key Laboratory of Molecular Developmental Biology, Institute of Genetics and Developmental Biology, Chinese Academy of Sciences, S2-316 Building #2, West Beichen Road, Chaoyang District, Beijing, 100101 China; 2 Zhengzhou Key Laboratory of Molecular Biology, Zhengzhou Normal University, Zhengzhou, 450044 China; 3 School of Life Sciences, University of Chinese Academy of Sciences, Beijing, 100049 China; 4 Beijing Key Laboratory of Captive Wildlife Technologies, Beijing Zoo, Beijing, 100044 China; 5 Beijing Zoo, No. 137 West straight door Avenue, Xicheng District, Beijing, 100032 China

**Keywords:** MHC, *Addax nasomaculatus*, BAC, Physical map

## Abstract

**Background:**

The mammalian major histocompatibility complex (MHC) harbours clusters of genes associated with the immunological defence of animals against infectious pathogens. At present, no complete MHC physical map is available for any of the wild ruminant species in the world.

**Results:**

The high-density physical map is composed of two contigs of 47 overlapping bacterial artificial chromosome (BAC) clones, with an average of 115 Kb for each BAC, covering the entire addax MHC genome. The first contig has 40 overlapping BAC clones covering an approximately 2.9 Mb region of MHC class I, class III, and class IIa, and the second contig has 7 BAC clones covering an approximately 500 Kb genomic region that harbours MHC class IIb. The relative position of each BAC corresponding to the MHC sequence was determined by comparative mapping using PCR screening of the BAC library of 192,000 clones, and the order of BACs was determined by DNA fingerprinting. The overlaps of neighboring BACs were cross-verified by both BAC-end sequencing and co-amplification of identical PCR fragments within the overlapped region, with their identities further confirmed by DNA sequencing.

**Conclusions:**

We report here the successful construction of a high-quality physical map for the addax MHC region using BACs and comparative mapping. The addax MHC physical map we constructed showed one gap of approximately 18 Mb formed by an ancient autosomal inversion that divided the MHC class II into IIa and IIb. The autosomal inversion provides compelling evidence that the MHC organizations in all of the ruminant species are relatively conserved.

**Electronic supplementary material:**

The online version of this article (10.1186/s12864-019-5790-2) contains supplementary material, which is available to authorized users.

## Background

Mammalian major histocompatibility complex (MHC) plays an indispensable role in host defence against infection by various pathogens [1]. Among jawed vertebrates, MHC is encoded by a highly polymorphic region organized into three clusters (classes I, II and III) [2]. MHC class I and class II encode molecules on the cell surface that present exogenous and endogenous antigens to T lymphocytes [3]. MHC class II molecules are further divided into classic MHC class II proteins (DQ, DR, and DP) and non-classic MHC class II proteins (DM and DO) depending on whether the binding to the antigen is direct [4]. MHC class III molecules consist of heat shock proteins and components of the complement system that can assist in immune responses [5]. In addition to antigen processing and presentation, MHC is also involved in genetic diversity and tissue transplantation, and may be involved in mate selection [6].

Research on MHCs of humans [7, 8], mice [9, 10], chickens [11, 12], pigs [13, 14], horses [15–17], dogs and cats [18], has revealed considerable conservation in structure and organization. In contrast to MHCs of humans and mice, ruminant MHC was divided in the class II region by a substantial autosomal inversion that divided class II into class IIa and IIb [19]. In addition, we noticed that *DY* is a newly evolved gene at the boundary of the autosomal inversion [20]; this gene is highly expressed in dendritic cells which are involved in the induction and function of regulatory T cells in the context of microbial exposure [21, 22]. The evolutionary significance of the autosomal inversion still remains largely unclear.

The addax (*Addax nasomaculatus*) is a critically endangered wild ruminant species listed on the International Union for Conservation of Nature (IUCN) Red List of Threatened Species in 2017 [23]. Previous studies on the addax have focused on the digestive tract [24, 25], parasites [26], microsatellite markers [27], and population genetics [28], while little is known about the addax MHC structure and organization. Although the correlation between MHC diversity and population viability is not fully understood [29], several studies have indicated that populations with limited MHC diversity may be vulnerable to emerging pathogens [30, 31] and that small populations are prone to lose functional alleles of MHC due to genetic drift and demographic fluctuation [32]. We thought it would be beneficial to construct an addax MHC map for the purpose of comparative mapping between wild and domestic ruminant species.

In traditional bacterial artificial chromosome (BAC) library construction, lymphocytes and liver tissue [33, 34] are used as sources of genomic DNA due to the relative ease of sample collection. However, considering the occurrence of somatic recombination and somatic hypermutation in immunoglobulin genes in the development of bone marrow-derived lymphocytes [35], BAC libraries from these cell types may not fully reflect true gene organization and characterization in the genome. Liver tissue has the same limitations considering that liver cells cannot be well separated from lymphocytes because of the rich blood supply in the liver [36, 37]. In addition, some studies have also reported that it is hard to assemble antibody-encoding regions in high-throughput sequencing due to their repetitive nature [38, 39]. Therefore, our addax BAC library, which used fibroblasts rather than blood cells or liver tissue as the genome source, could reveal the intact genomic structure and facilitate contig assembly, especially within antibody loci, during genome sequencing.

In this study, based on the construction of an addax BAC library from fibroblast cells, we constructed a high-density physical map of the addax MHC region consisting of 47 overlapping BAC clones with only one gap located between MHC class IIa and class IIb. The order of the BAC clones which covered addax MHC region was confirmed by DNA fingerprinting, and the overlapping relationship was cross-checked by BAC-end sequencing and sequence-specific PCR. This physical map could facilitate the protection of endangered addax and enhance understanding about ruminant MHC evolution.

## Methods

### Ethics statement

The primary cell line used for addax genomic DNA isolation was maintained in the laboratory of Professor Runlin Z. Ma. The skin sample of addax used to establish the primary cell line was obtained from an accidently injured male addax maintained in the Beijing Zoo. The animal was anesthetised during the procedure. The project was a research collaboration between the Beijing Zoo and the Institute of Genetics and Developmental Biology (IDGB), Chinese Academy of Sciences, and the consent was obtained from the appropriate authorities at the zoo. Ethics approval was obtained from the Animal Care and Use Committees in IGDB, with the approval number AP2016054.

### BAC library construction

The addax primary fibroblast cell line was established by explant culture using a small piece of addax skin as the initial source. The skin tissue-derived fibroblast cells were embedded in 1% low-melting point agarose (Sigma Aldrich Co., MO, USA) at a concentration of 5 × 10^7^ cells /ml to form plugs for subsequent genomic DNA isolation. The DNA plugs were subjected to brief electrophoresis to remove small DNA fragments following proteinase K digestion. After partial digestion with *Hin*dIII (New England Biolabs, MA, USA), the large DNA fragments in the plugs were visualized in 1% TAE agarose gels following pulsed-field gel electrophoresis (PFGE). DNA fragments in the 100–300 Kb range were collected and ligated into a commercial BAC vector (pCC1BAC). The ligation mixture was electro-transferred into commercial electro-competent *E. coli* (Epicentre, WI, USA) after desalting in a 1% agarose cone. The transformed *E. coli* cells were cultured at 200 rpm for one hour before plating on LB agar plates containing chloramphenicol, IPTG and X-gal. White colonies were individually transferred into 384-well plates for growth, storage, and replication. Each 384-well plate had two replicate plates that were kept in a − 80 °C freezer for long-term storage.

### BAC library characterization

To evaluate the quality of the constructed addax BAC library, a total of 172 random BAC clones were picked from the LB plates for insert size determination, as well as determination of the proportion of empty BAC vectors. Individual plasmid DNA was purified from each of 172 BAC clones and subjected to restriction enzyme digestion using *Not*I (New England Biolabs, MA, USA). The digestion products were visualized by ethidium bromide in 1% TAE agarose gels following PFGE.

### Identification of MHC-positive BAC clones

MHC-positive clones were identified from the addax BAC library by PCR-screening of the library using consensus PCR primers designed with bovine and ovine MHC sequences as templates. Bovine and ovine MHC sequences were acquired from the NCBI website (https://www.ncbi.nlm.nih.gov/genome/?term=bos+taurus, https://www.ncbi.nlm.nih.gov/genome/?term=ovis+aries). Primers were designed along the MHC region approximately 50–80 Kb apart using the software Oligo7 (Molecular Biology Insights Inc., CO, USA). All primers were custom-synthesized by Shanghai Sangon (Sangon, Shanghai, China). A total of 51 primer pairs were designed for screening the addax BAC library (Table 1). The addax BAC library of 500 plates was organized into 50 super-pools for screening efficiency. After the potential MHC-positive clones were determined in a certain super-pool, screening was continued for 3-dimensional BAC clone pools of plates, rows, and columns for large-scale PCR screening based on the methods of Li [40]. The PCR products were analysed by 1% TAE agarose gel electrophoresis, and clones with the expected size were picked for subsequent verification.

**Table 1:** Comparative primers used for the identification of positive BAC clones in the MHC region^a^

Name	Gene symbol	Primer sequence (5′ → 3′)	Product (bp)	Positive addax BAC clones
S0	*MOG*	F: TGCATGCTGAGACTACACCA	572	97H9;427 M6
		R: CTGAACTAGGACAGTGCAGG		
S02	*PPP1R11*	F: AGAACCGGAGCCTAACCATC	641	97H9;427A11; 372A2
		R: CCGCACACAGTGTGTATGAC		
S03	*TRIM10*	F: ATGTGTGTGACCATGAGGCT	650	427A11;470I19
		R: TCAGTCTAGTCGCATCCGAG		
S04	*TRIM26*	F: CCTTGCATGAAGTCAGCTTG	919	470I19
		R: CCACCTCTGAGCTGAATGAA		
S05	*OLA-I*	F: ACACCGCACTCGGCTACTAC	332	273H17;419 N6; 493 K20
		R: GCGATATAATCTCTGCCGTC		
S06	*LOC101110710*	F: AGATCTCCAAGCTCAAGCCT	805	none
		R: TGTGTGTCTGACAGCTGCGA		
S07	*ABCF1*	F: GCGAAGCCAGAGTTACCATA	1119	427P1;392 L11; 51E6
		R: CCACAGCATTCTCAAGCACA		
S08	*ATAT1*	F: ATAGTGTGAAGGCCATGAGG	908	427P1;427H1; 397 K7
		R: GCTCAAGTTCTTGGATCGGT		
S09	*NRM*	F: GCTTGAGAGTGCCTTGGTAA	514	350H17
		R: ATGAGCTCTGCGTAGTCGAA		
S10	*FLOT1*	F: GCACAGAAGTTGTCAGCACC	911	350H17;297P6
		R: TCGTCATTCTCTCTGGAAGC		
S11	*DDR1*	F: GACTCCATGTTGTGCAGGCT	1114	97P6;206A17; 132D20
		R: GTCCTCACCTGGCATTCTTG		
S12	*DPCR1*	F: CTAACATCACAGAGGCAAGC	1129	none
		R: TAAGTGAATGTGTGCGGAGC		
S13	*CDSN*	F: CAGTAACGCTGTGAGGAAGG	830	395 M8;132D20; 205C9
		R: GAGATGGCAGCTTATTCTGG		
S14	*POU5F1*	F: CTCGGACCTGGATGAGCTTC	1256	345 K6;395 M8
		R: TATTCAAGGCTCAGCAGTCG		
S15	*MICA*	F: AACTCCTGGCAGAGATCTTG	1064	345 K6;368L6
		R: CTCACTGTCCACTTCTGGCT		
S16	*LOC101105609*	F: CTCAACAGAGTTCGCGATCA	851	none
		R: AAGCTAGAGTCTGGCCTCCT		
S17	*MCCD1*	F: GGCTCACCAGTTCAACCAAG	747	217P17
		R: CACTGCGTGTAGAGCTCGTC		
S18	*TNF*	F: GGAACCAGAGGATAAGCTGA	980	217P17;269 L19
		R: TCATCTGGAGGAAGCGGTAG		
S19	*APOM*	F: ACGACGCCAATCACAGTAAG	986	145D13
		R: CTCAACGAGGACGATGGTAA		
S20	*LY6G6C*	F: GGATGTGGTTCACCTGGTCA	904	493O11
		R: CGTGGAGGTTACACACTTGC		
S21	*VARS*	F: CTTGGACTGGAATCGAGCCT	839	none
		R: AGCCTTGGACCTTGTAAGCA		
S22	*HSPA1A*	F: CTACGTGGCCTTCACCGATA	1214	493O11;383F23
		R: CCTCGTACACCTGGATCAGC		
S23	*C2*	F: TGTGATAATCGTCCAGCTCC	1549	299D13
		R: CCGATGGCATAGATGTCTGA		
S24	*STK19*	F: GGATTGTCCAGCTAGGCTTC	1182	none
		R: CCTACACACACCTCACCACG		
S25	*TNXB*	F: CCAGATGTGCATACCACTCA	942	218D15;120D3
		R: GGTGTGCTCAGCATCTTCCT		
S26	*PBX2*	F: GATAATGACCATCACCGACC	1074	279C4;500O21
		R: CCAGAGTTGAGGAATCGGAG		
S27	*NOTCH4*	F: TCCTGGTACTCCATTCTTGC	1006	279C4;500O21
		R: GATTGACACGCGTCTGGTTC		
S28	*LOC101111058*	F: ACCAGGTGACTGTCCAGAGG	1117	53B21;301E21
		R: GTGAAGTACAGGCCAGCTCC		
S29	*LOC106991808*	F: GCTGCCCATCTGTCCACGA	658	485B7;430H19
		R: AATTCAGTTGAAAGGCACACT		
S30	*C20H6orf10*	F: CACACGTACCTTCGGCTCT	616	415H14;82H18; 55D19
		R: ATTCTTCTGTCCACGCACT		
S31	*LOC105603755*	F: TTCCCTTTCAGGTTCTCACCA	441	390I7;292P1
		R: TGCACAGTTATGATTGTTGGAC		
S32	*LOC105603754*	F: AGCTTCTTTGTGAAAACGCAT	499	424B10;146 M21
		R: GCTATTCCCCGTGGATACCAAA		
S33	*LOC101110546*	F: CTTTGGATACAGTTACGCTCCT	944	392G17;478N22
		R: ACCAATGAAACAGAATAGAGCC		
S34	*LOC105603751*	F: GAAGGCGTCAGTCCATACCC	707	none
		R: ACAGAGCCTTATGGTTCCGAA		
S35	*LOC101110277*	F: GCCTCATCCGACAGCACCG	795	420O12;368E9
		R: CCTTTCCTTGTGCTACGCTT		
S36	*BTNL2*	F: CCTCTCGCTCTGCTATGGTT	861	473F22;473H22; 233P9
		R: CAGAAGGCATGCACCACTTA		
S37	*LOC101120871*	F: GGTGAACACGGTGTGCAGAT	907	244 M7;392G17
		R: ACCATGAGTTGTGCAGCTGA		
S38	*DQA*	F: CAGGAGTATTGCTGAGCAGG	1274	244 M7;247O24
		R: TGACAAGGACAGTGGCAATG		
S39	*OVAR-DRB3*	F: GACAGGAGCTAGATTGGACC	924	1 M15;372C2
		R: CCATGACAGCCTGCACATAC		
S40	*LOC101120118*	F: AGGCAACGTCCAATGGTACT	1321	385F12;381B3
		R: CATGAATCCGCTGCATTCTC		
S41	*LOC101109220*	F: ATGATGACATCTGGCACAGG	970	385F12;401K6
		R: CAACAGAGGTATGAGCCAGG		
S42	*OVAR-DRB1*	F: CCTGTAATGGAAGTCCTCTG	1272	432 J13;134D8
		R: GCCAACTTGCTCTCTATGCT		
S43	*ELOVL5*	F: CAGTGCATGGACCTACGAAC	1562	134D8;276 J2
		R: CAGGAGGCAGATCTACATGA		
S44	*GCM1*	F: TTGGAAGAGCTAGGAAGCAC	1151	276 J2;466O4
		R: CTCGAGTCACCAGATCCTTG		
S45	*VPS52*	F: CGCTTGAAGGATTGGTGACT	930	444E20
		R: GACCATGATCTGCACTCAGG		
S46	*RXRB*	F: TGTACAGGAGGAGCGTCAGC	973	354 J10
		R: GGTGAGGTCAACGAGACTGG		
S47	*LOC101110545*	F: TCTTCTCATCTCGCACACAG	982	none
		R: GAAGAAGCAGGCCTTGAATC		
S48	*BRD2*	F: CTGTATACTCCACCGCCTGA	900	270A14
		R: GTGTGTGTGGCCTCCAACTA		
S49	*PSMB9*	F: ACCAGATAACCTTGAGCCAG	869	176F14
		R: AGGATCATACAGCGAGTTGC		
S50	*DYA*	F: CGAGCTCTTCTACGTGGACC	1037	94B2
		R: GACCACAGCTTCCTCGTTCT		
S51	*GCLC*	F: TGATGGAACATCAGGCCTAC	841	68 K22;427 K6
		R: GAGAAGGCGTCCGACTATAA		

^a^The comparative primers were designed with bovine and ovine MHC consensus sequences as templates. A total of 51 pairs of primers are listed here

### DNA fingerprinting of the identified BAC clones

To determine the overlap of the potential MHC-positive clones, each BAC plasmid was completely digested by the restriction enzyme *Hin*dIII at 37 °C for 8 h. The digested products were subjected to PFGE on 1% TAE agarose gels. Fingerprinting images were captured with a UVP LabWorks system (UVP Inc., CA, USA). The restriction fragment patterns were analysed to identify overlapping BAC clones, which were then manually assembled into draft contigs based on the modified methods of Marra [41] and Soderlund [42]. Contigs were assembled from the fingerprint data using the computer program FPC (http://www.agcol.arizona.edu/software/fpc/) with a tolerance of 3 and a cutoff of 1e-10.

### BAC contig assembly

Guided by the order and location of the comparable bovine and ovine MHC loci, BAC contigs covering the entire addax MHC region were assembled by integrating the results from DNA fingerprinting, sequence-specific PCR, and BAC-end sequencing. There were two sequencing primers residing at restriction enzyme sites in the pCC1BAC vector that could be used to identify insert sequences at both ends by Sanger sequencing. Gaps in the contigs were closed by several rounds of successive PCR screening and by DNA fingerprinting of additional BAC clones. Overlap between adjacent BAC clones was further verified by PCR amplification of the shared DNA fragments between the clones and by final verification through DNA sequencing of the fragments. The oligonucleotide primers used for the DNA sequencing were a CopyControl pCC1BAC vector-derived T7 sequencing primer (5′-TAATACGACTCACTATAGGG3’), pCC1/pEpiFOS RP-2 (abbr.RP-2) (5′-TACGCCAAGCTATTTAGGTGAGA-3′), and pCC1/pEpiFOS RP-1 (abbr.RP-1) (5′-CTCGTATGTTGTGTGGAATTGTGAGC-3′). The acquired BAC-end sequence was used to analyse overlap and to design primers for sequence-specific PCR (Additional file 1: Table S1).

## Results

### Characterization of the addax BAC library

**Fig. 1:**
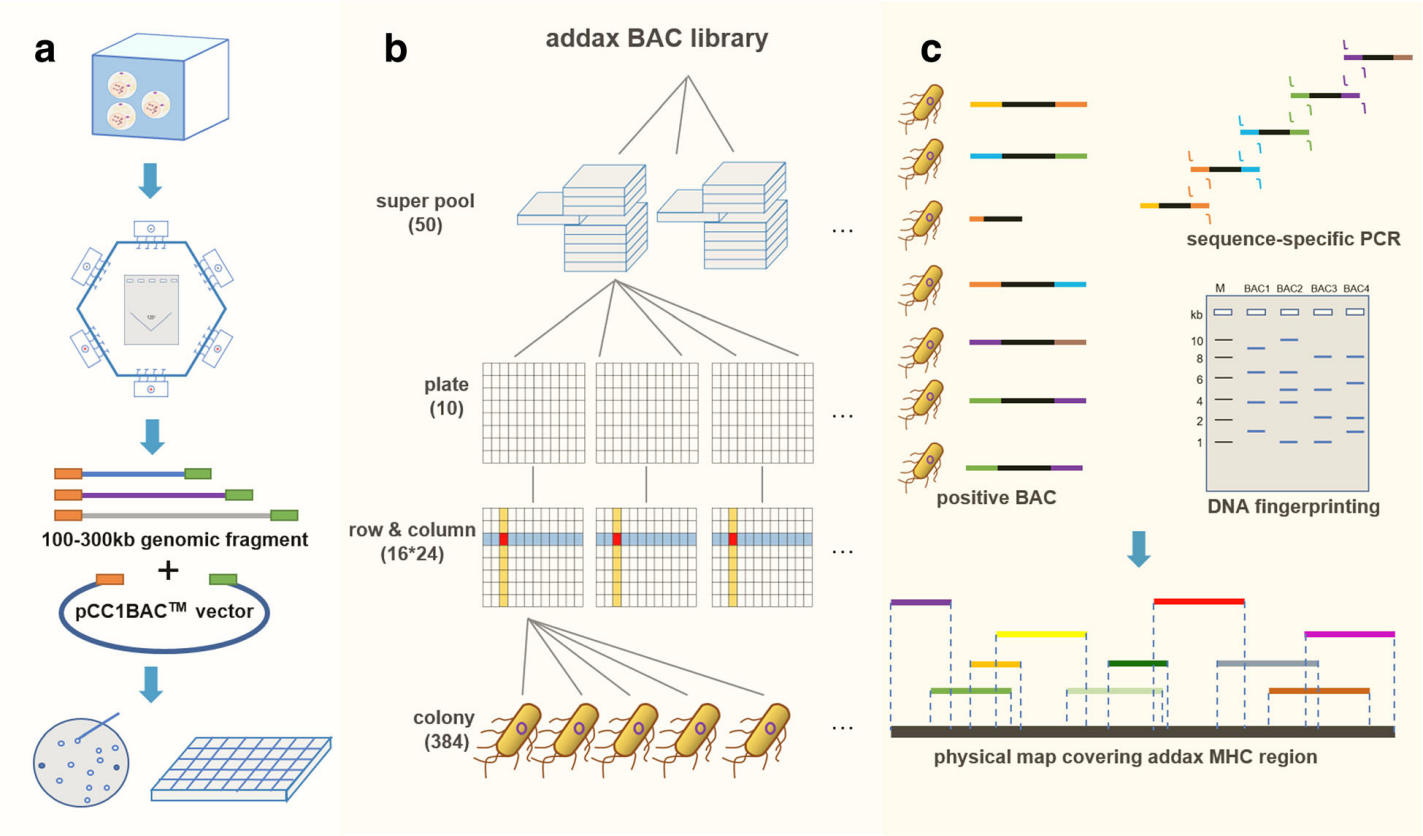
Overview of the addax MHC physical map construction. **a** BAC library construction. Fibroblasts were embedded into low-melting point agarose to form plugs for subsequent genomic DNA isolation. Genomic DNA fragments of 100–300 Kb were ligated into the pCC1BAC vector. White colonies were picked into 384-well plates after transformation. **b** 3D-PCR screening strategy. To improve screening efficiency, the entire addax BAC library composed of 500,384-well plates was divided into 50 super-pools. After the positive MHC clones were determined in a certain super-pool by the first dimension of PCR, the second dimension of PCR was further performed on 10 plates. Then, the third dimension of PCR screening was continued on 16 rows and 24 columns. The intersection of a row and column indicated a potential positive clone covering the addax MHC region. **c** The physical map covering the addax MHC region was assembled by integrating the results from sequence-specific PCR and DNA fingerprinting

**Fig. 2:**
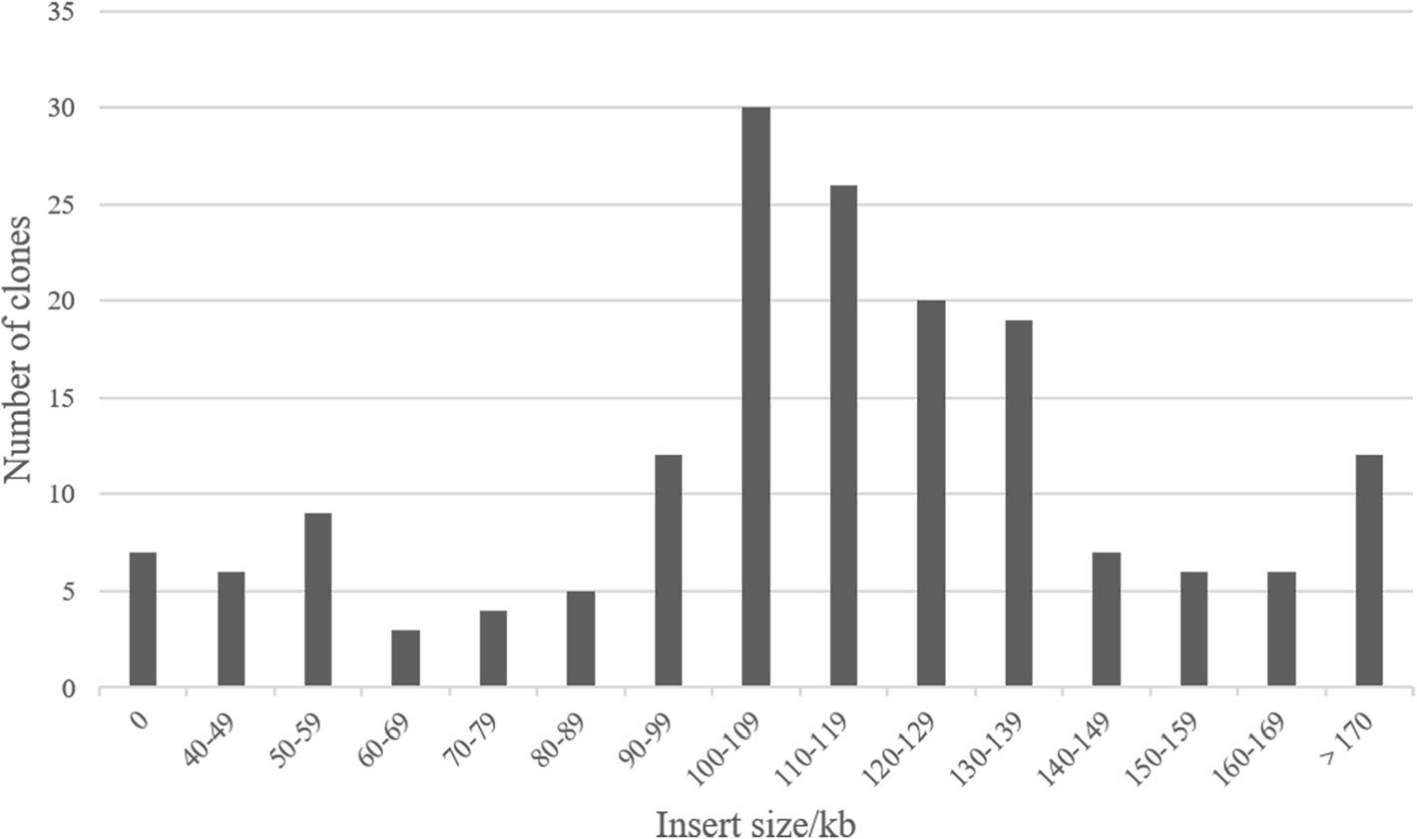
Distribution of insert sizes in BAC clones of the addax (*Addax nasomaculatus*). A total of 192,000 BAC clones were harvested and stored in 500,384-well plates. Based on an analysis of 172 random BAC clones, the average insert size of the genomic DNA in a BAC was approximately 115 Kb, and the insert size for over 80% of BAC clones was larger than 90 Kb. The percentage of empty vectors was less than 5%

A graphic overview summarizing our workflow is shown in Fig. 1. We successfully constructed an addax BAC library composed of approximately 192,000 clones with an average insert size of approximately 115 Kb (Fig. 2). The BAC library we constructed was of excellent quality, as indicated by an empty-vector rate of less than 5% (Fig. 2). According to the Animal Genome Size Database, the C value of the addax is 3.98, as estimated by flow cytometry [43]. Therefore, this library should contain 5.4-fold genomic equivalents. This library could serve as a genetic resource in zoo-fluorescence in situ hybridization (Zoo-FISH) for MHC comparative genomic research and provide genomic content information for other chromosome regions.

### Positive BAC clone screening

Based on bovine and ovine MHC consensus sequences, we designed a total of 51 pairs of PCR primers to screen the addax BAC library. First, potential MHC BAC clones were determined in a certain super-pool by PCR. Then, the location of MHC BAC clones was acquired by performing PCR in 10 plates, 16 rows and 24 columns with a method developed by Li [40]. A total of 68 MHC-positive clones were identified by 44 pairs of primers derived from bovine and ovine MHC consensus regions, which generated products of the expected size (Table 1). The relatively high efficiency in the screening process indicated the homologous natures of the addax, bovine, and ovine MHC regions.

### Validation of the overlap between adjacent BAC clones

**Fig. 3:**
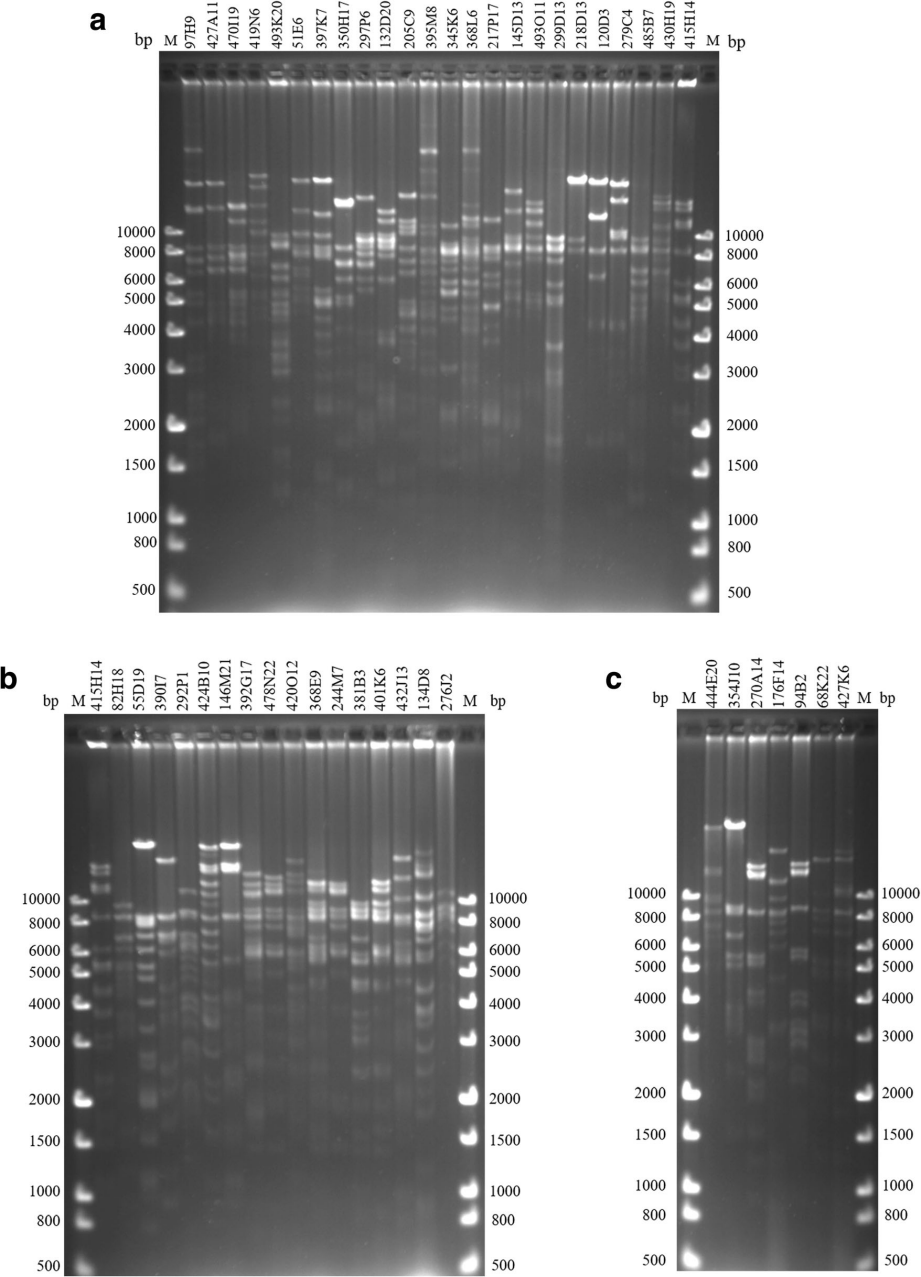
DNA fingerprintings of the 47 positive BAC clones to determine the overlap. The positive BAC clones identified in the PCR screening were digested with *Hin*dIII and then separated on a 1% TAE agarose gel. The gel was stained with ethidium bromide (EB) for imaging with a UVP LabWorks system. M: DNA size standard marker (1 Kb Plus DNA Ladder from Transgene); the base pair (bp) sizes are indicated on both sides. **a**, **b** Fingerprints of BAC clones consisting of addax MHC contig 1. **c** Fingerprints of BAC clones consisting of addax MHC contig 2

**Fig. 4:**
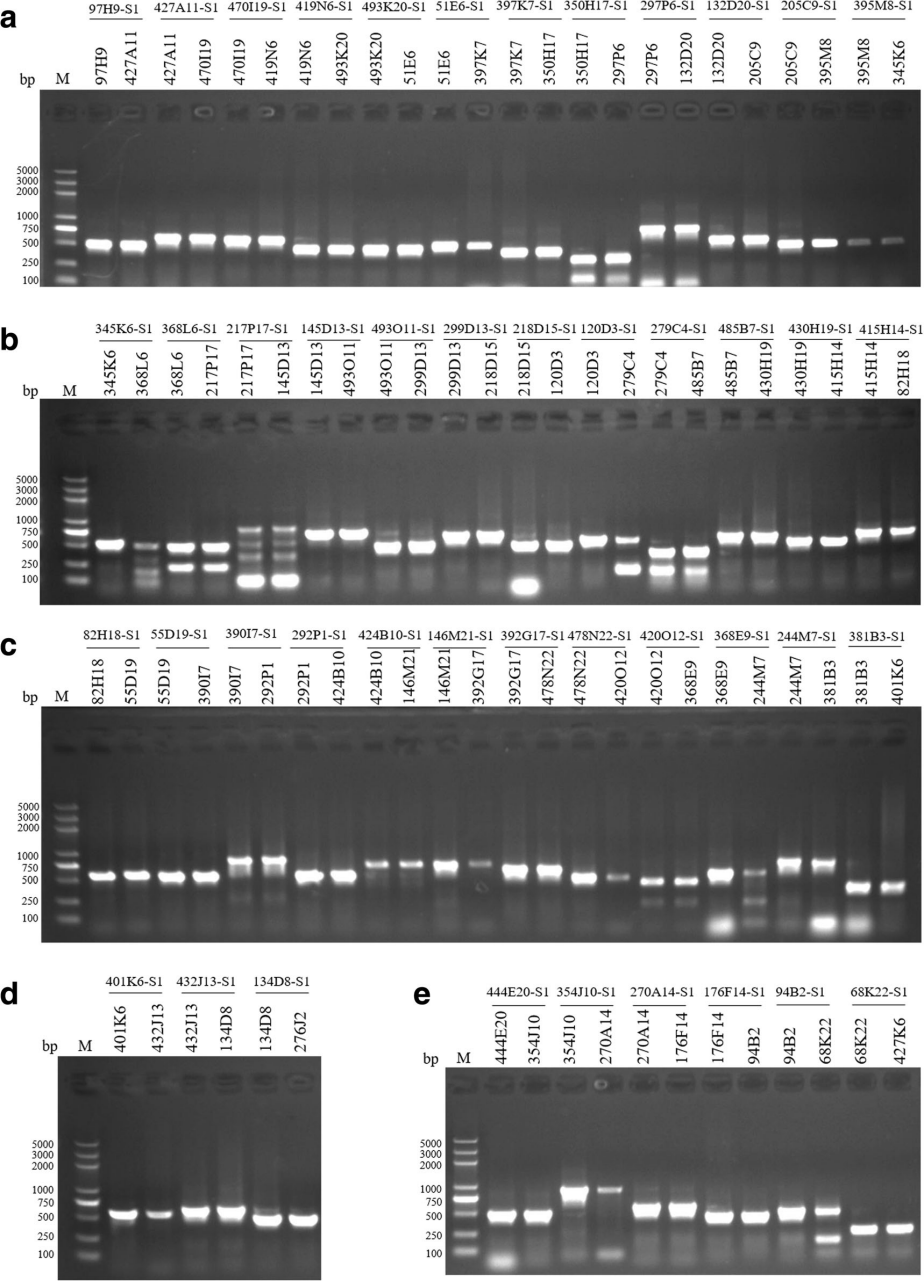
PCR verification of the overlap between pairs of overlapped BAC clones. Pairs of overlapped clones were PCR-amplified using the same primer pair designed based on the BAC-end sequence. The markers above the black lines indicate the primer pairs, and the ones below the lines are labels of positive clones used as PCR templates. M: DNA size standard (DL2000 Plus DNA Ladder from Transgene); the base pair (bp) sizes are indicated on both sides. **a**-**d** Validation of the overlap between clones covering the addax MHC class I–class III-class II a region. **e** Validation of the overlap between clones covering the addax MHC II b region

To determine the overlap of the BAC clones, plasmid DNA from 68 of the selected MHC-positive BAC clones was fully digested by the *Hin*dIII restriction enzyme, and the enzyme products were separated by PFGE for DNA fingerprinting. Analysis of the DNA fingerprinting patterns (Fig. 3) helped to identify BACs with overlap, as evidenced by sharing more than 2 identical *Hin*dIII fragments. Redundant BAC clones were removed during the extension of the BAC contig. The overlap of the BACs was further verified by DNA sequencing of the sequence-specific PCR products, whose primers were designed according to BAC-end sequencing (Fig. 4). A total of 45 out of 50 pairs of PCR primers designed for sequence-specific PCR generated the expected fragment size between two overlapping clones (Additional file 1: Table S1). Sequence-specific PCR, which served as a cross-check method, enhanced the accuracy of the identification of overlap between adjacent clones.

### Assembly and characterization of addax BAC contigs

We successfully constructed an addax MHC physical map using a total of 47 positive BAC clones selected from the BAC library. After cross verification by both BAC-end sequencing and co-amplification of identical PCR fragments within the overlapping region, 47 BAC clones were ultimately chosen to construct the minimal tiling path of the addax MHC region. DNA fingerprinting consisted of 47 BAC clones covering the whole addax MHC region (Fig. 3). The insert sizes of the 47 BAC clones were analysed to support information about the size of the addax MHC region (Additional file 2: Figure S1). The first contig had 40 overlapping BAC clones (Fig. 3 a, b), covering an approximately 2.9 Mb of genomic regions including the entire MHC class I, class III, and class IIa. *DR* and *DQ* loci were localized to the MHC class IIa region. The second contig had 7 BAC clones (Fig. 3c), covering an approximately 500 Kb genomic region that harbours MHC class IIb. *DM*, *DO* and *DY* loci were found in this region. All clones are available for public cross-checking.

**Fig. 5:**
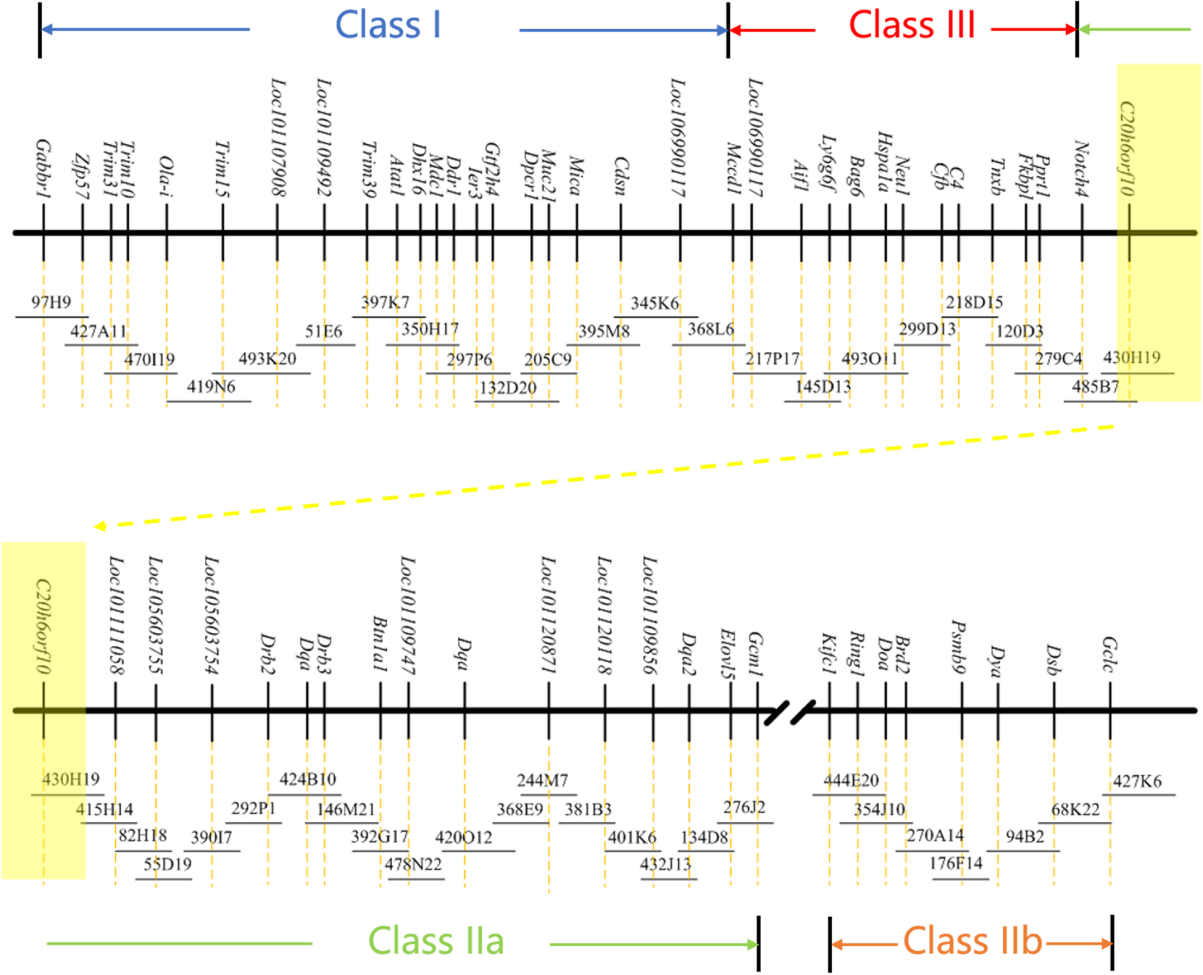
Physical map covering the entire MHC region of the addax**.** The order and orientation of BAC clones (overlapping horizontal bars with clone ID name listed above) were determined based on integration of the results of DNA fingerprinting, BAC-end sequencing, and sequence-specific-PCR. The genes identified by BAC-end sequencing are marked by vertical bars along the horizontal line, with the locus names listed above. The continuous BAC map is represented by two panels with the overlapping regions marked with the same coloured shadows at the ends

A complete physical map of a BAC clone contig covering the addax MHC region was successfully assembled (Fig. 5) based on the integrated results of DNA fingerprinting, BAC-end sequencing, and sequence-specific PCR of the BAC ends. The gene order and organization of the addax MHC are well conserved compared with the ovine MHC (Additional file 3: Figure S2). The addax MHC physical map we constructed thus far has only one gap, which was caused by an autosomal inversion that divided the MHC class II into class IIa and IIb. However, such an autosomal inversion provides compelling evidence that the MHC organization in ruminant animals are relatively conserved.

## Discussion

In this article, we presented a novel MHC physical map for the addax that spanned approximately 3.4 Mb. The two contigs, which covered the whole addax MHC region, spanned approximately 2.9 Mb and 500 Kb genomic regions, respectively. This is the first comprehensive physical map of the MHC of addax. This study provides a preliminary foundation for better estimating addax MHC diversity, which could help to avoid future inbreeding that would potentially impair immune function in the population [44, 45]. Therefore, this physical map provides an important knowledge for the study and protection of the addax.

Based on the comparative gene map of the MHC region among cattle, sheep, and addax (Additional file 3: Figure S2), the organization of the addax MHC is more homologous to that of the ovine MHC than to that of the bovine MHC, which is consistent with the phylogenetic relationship of Hippotraginae being closer to Caprinae than to Bovinae [46]. There is only one gap in this physical map located in MHC class IIa and IIb. According to NCBI genomic data, the autosomal inversion in the sheep genome is approximately 18 Mb. Therefore, the addax gap is probably close to 18 Mb because of the high similarity between the addax MHC and the ovine MHC.

According to bovine and ovine genomic data [47, 48], the bovine and ovine MHC regions span approximately 3.9 Mb and 3.1 Mb, respectively. The addax is a species of Hippotraginae, and the length of its MHC region is between cattle (subfamily Bovinae) and sheep (subfamily Caprinae). These discrepancies may be due to variations in the number of class I genes among cattle, sheep, and addax [49]. It has been proposed that individuals of wild populations possess more MHC-DRB alleles than individuals of captive populations [50]. These differences in MHC region size may be the result of wild and domestic animals experiencing different evolutionary selective pressures.

This physical map of the addax MHC provides additional evidence for the hypothesis of ruminant ancestral chromosome rearrangement [51, 52]. This hypothesis has been supported through comparison of bovine and ovine MHC sequences with MHC sequences from other non-ruminant species, such as humans, chimpanzees and mice [40]. Our results suggested that at least in Bovidae, an inversion occurred in the MHC class II region and autosomes, resulting in a discontinuous MHC class II region and autosomal inversion. In addition, a characterization of the MHC class II region in the Yangtze finless porpoise indicated that its MHC class II region was also divided into two segregated sub-regions [34]. These findings suggested that the inversion in MHC class II may be prevalent in cetaceans and ruminants.

## Conclusions

We have successfully constructed a high-density physical map covering the entire MHC region of the addax. A total of 47 effective BAC clones were selected by comparative PCR screening to constitute this physical map. This is the first complete MHC physical map of wild ruminants that could provide valuable information about addax protection and facilitate our understanding of ruminant evolution.

## Additional files

Additional file 1: **Table S1.** Sequence-specific PCR primers used for validation of the overlap between BAC clones covering the addax MHC region. A total of 45 pairs of primers are listed in this table. (DOCX 21 kb)

Additional file 2: **Figure S1.** Distribution of insert sizes in the BAC clones covering the addax MHC region. (DOCX 751 kb)

Additional file 3: **Figure S2.** Schematic (not to scale) comparing genomic organization of the MHC region in cattle, sheep and addax. The blue, red and green bars represent genes in the MHC class I, III, and II regions, respectively. The bovine and ovine gene maps were adapted from Ensembl and NCBI annotations (not all annotated genes are shown here). (DOCX 363 kb)

## Abbreviations

BAC: Bacterial artificial chromosome
IUCN: International Union for Conservation of Nature
MHC: Major histocompatibility complex
PCR: Polymerase chain reaction
PFGE: Pulsed-field gel electrophoresis
Zoo-FISH: Zoo-fluorescence in situ hybridization
