# Supplementary material for: A high-density BAC physical map covering the entire MHC region of addax antelope genome
Source: BMC Genomics. 2019 Jun 11;20:479. doi: 10.1186/s12864-019-5790-2 (PMC6558854; doi:10.1186/s12864-019-5790-2)
Supplement: Supplementary file 1 — Table S1. Sequence-specific PCR primers used for validation of the overlap between BAC clones covering the addax MHC region. A total of 45 pairs of primers are listed in this table. (DOCX 21 kb) [file 12864_2019_5790_MOESM1_ESM.docx]

**Table S1** Sequence-specific PCR primers used for validation of the overlap between BAC clones covering the addax MHC region. *

| **Name** | **Primer sequence (5'-3')** | **Product**  **(bp)** | **Ann. T. (℃)** | **BAC Clone Verified** |
| --- | --- | --- | --- | --- |
|  |  |  |  |  |
| 97H9-S1 | F: TTTTCACGGAACAGCTATGACC | 511 | 55 | 427A11 |
|  | R: TGAGCCACCAGAGAAGTCCT |  |  |  |
| 427A11-S1 | F: ACCATGATTACGCCAAGCTA | 632 | 56 | 4701I9 |
|  | R: GGACCGGCCAAATTCAATGC |  |  |  |
| 470I19-S1 | F: GGCATGCAAGCTTCTTACCCGA | 613 | 60 | 419N6 |
|  | R: AGGTGCTGTGCTCTGTCTGT |  |  |  |
| 419N6-S1 | F: CACCTTTCATGGCCGCAGA | 425 | 57 | 493K20 |
|  | R: AGAAGGAAATGGCAACCCAC |  |  |  |
| 493K20-S1 | F: CACGGAACAGCTATGACC | 430 | 55 | 51E6 |
|  | R: CCCCAATACTACGACCAG |  |  |  |
| 51E6-S1 | F: CCCCTCAACAGCACGAGCGAAG | 456 | 58 | 397K7 |
|  | R: AGACTACAGCCCAGCATCGG |  |  |  |
| 397K7-S1 | F: ACCGGCCTTTCCCCTTGACT | 414 | 60 | 350H17 |
|  | R: AGCCCCTGAAGCACCGAGACA |  |  |  |
| 350H17-S1 | F: ACGACTCACTATAGGGCGAAT | 366 | 56 | 297P6 |
|  | R: ACAGGCAGGTTCTTTACCAC |  |  |  |
| 297P6-S1 | F: ATTTCCCCACCATGGACCCT | 745 | 58 | 132D20 |
|  | R: TGCAAATGCGGGACAATTGACA |  |  |  |
| 132D20-S1 | F: ACGGGAAACAGCTATGACCA | 510 | 54 | 205C9 |
|  | R: TGATAGGCACTCAGGTCCAA |  |  |  |
| 205C9-S1 | F: GCAGCCCCACGCCCACTC | 487 | 62 | 395M8 |
|  | R: CCCCTGTGTTTGCGTGTCATCC |  |  |  |
| 395M8-S1 | F: ACCTGTAGGAGCAAGCTTCCC | 490 | 58 | 345K6 |
|  | R: TTGTGCGTGTGTCCACTCCC |  |  |  |
| 345K6-S1 | F: GGATCCTCTAGAGTCGACCTG | 505 | 56 | 368L6 |
|  | R: TGCTCAGAAGCTGTTTCCCC |  |  |  |
| 368L6-S1 | F: ATGTTGGGCTCCTCAGACAGT | 497 | 61 | 217P17 |
|  | R: CCAAGCCCTCCTCCAGCGACA |  |  |  |
| 217P17-S1 | F: TTGATAGACAGACCACCGAA | 882 | 53 | 145D13 |
|  | R: ACACTGTTAATTTCTTGCCAT |  |  |  |
| 145D13-S1 | F: CCCACCCGTCCTGATTCCG | 736 | 58 | 493O11 |
|  | R: CCTGAGCCTGCAAGTACCCAT |  |  |  |
| 493O11-S1 | F: ATCAGACCCATGTACCCTGC | 452 | 55 | 299D13 |
|  | R: GCATCACCGACTCGATGGAC |  |  |  |
| 299D13-S1 | F: CACCGGAACAGCTATGACCA | 550 | 58 | 218D15 |
|  | R: AGGCTTCCAAGGTCAACCAC |  |  |  |
| 218D15-S1 | F: CCCCTGAACAGAAAGACATGC | 443 | 57 | 120D3 |
|  | R: AGTTGTACTGCCAATCTAACCC |  |  |  |
| 120D3-S1 | F: AGCCCTACTACCTCCCAACGAC | 495 | 60 | 279C4 |
|  | R: GCCTCCTCTAACCCCGGCAT |  |  |  |
| 279C4-S1 | F: ACGCCAAGCTATTTAGGTG | 341 | 54 | 485B7 |
|  | R: CAAACGGAAGAAGACCTG |  |  |  |
| 485B7-S1 | F: AAACAAAGAATAGAATGGTGCT | 572 | 55 | 430H19 |
|  | R: CCCCGTCCCATTCTAAAGGA |  |  |  |
| 430H19-S1 | F: GAGCAACAGCTATGCACCA | 425 | 56 | 415H14 |
|  | R: TTCCCCTATGTTCCATTCCC |  |  |  |
| 415H14-S1 | F: CTGTGGCCTTCCAAGCTC | 635 | 53 | 82H18 |
|  | R: TCCTTCAAATGGCAATCAACCC |  |  |  |
| 82H18-S1 | F: TTCACGGAACAGCTATGACCA | 592 | 54 | 55D19 |
|  | R: TTGTTTGTGGCCTCCCGACC |  |  |  |
| 55D19-S1 | F: ACGGAACAGCTATGACCA | 564 | 54 | 390I7 |
|  | R: AAGTTCAAGACCCACCGTT |  |  |  |
| 390I7-S1 | F: TTTCCCAGTCACGACGTT | 831 | 53 | 292P1 |
|  | R: AATGTTGCTGTATAGCCCTC |  |  |  |
| 292P1-S1 | F: CAGCCAACAGGTAATGCCCTT | 575 | 58 | 424B10 |
|  | R: CCAGGCTCCCTCTGTCCATG |  |  |  |
| 424B10-S1 | F: TTTTCCAGTCACGACGTT | 762 | 52 | 146M21 |
|  | R: CTTTTCTGATACAATCTAGGCA |  |  |  |
| 146M21-S1 | F: GCTATGACCATGATTACGCCAA | 760 | 52 | 392G17 |
|  | R: CTCCTTTCCTGAGGTGCTT |  |  |  |
| 392G17-S1 | F: TTTGCCTGCGTATAAACCCT | 630 | 56 | 478N22 |
|  | R: ACTGAGAAAGCCATCAAGGACA |  |  |  |
| 478N22-S1 | F: AGTTTGAAAAGTCAGGCATC | 459 | 52 | 420O12 |
|  | R: GAGGTCTTTCATAAGTGCAT |  |  |  |
| 420O12-S1 | F: AACCACATTCCTCTAGCCTA | 440 | 54 | 368E9 |
|  | R: AGAAAAGTATCCCCTTACCCT |  |  |  |
| 368E9-S1 | F: TAGAGTCGACCTGCACGCAT | 465 | 58 | 244M7 |
|  | R: TTGATCCCCTGCGTTCAGCC |  |  |  |
| 244M7-S1 | F: CACGGAACAGCTATGACC | 737 | 53 | 381B3 |
|  | R: CTTCCAGGCATGTATTACAAC |  |  |  |
| 381B3-S1 | F: CCCAAAGAAGACCCCAGACCTG | 338 | 63 | 401K6 |
|  | R: TGGCATCAGCAAGGCACACT |  |  |  |
| 401K6-S1 | F: GCCAGTGAATTGTAATACGACT | 539 | 53 | 432J13 |
|  | R: GCCAGCAATCTTGATTCTAGC |  |  |  |
| 432J13-S1 | F: TCCGCTTTCTCTTCTCCGAAC | 550 | 56 | 134D8 |
|  | R: GCCTCAGCCTTCAACCAC |  |  |  |
| 134D8-S1 | F: ACTCCCAGTAAATACCCGACT | 460 | 60 | 276J2 |
|  | R: AAGCCAACACAAGGGGTCTC |  |  |  |
| 444E20-S1 | F: AGGTCACAATTCCGGGTCCTT | 476 | 62 | 354J10 |
|  | R: ACTACTTGAGCTTTGTGCCCTT |  |  |  |
| 354J10-S1 | F: CCACGGAACAGCTATGACCA | 991 | 58 | 270A14 |
|  | R: TGCACAACGCGACCCAGT |  |  |  |
| 270A14-S1 | F: GCCAGTGAATTGTAATACGACT | 545 | 55 | 176F14 |
|  | R: ACATGGGCTTTCACAACAGG |  |  |  |
| 176F14-S1 | F: ACGACTGAAGCGACTTAGCA | 443 | 53 | 94B2 |
|  | R: TTTCCCCTTGAGTGTAATCAGC |  |  |  |
| 94B2-S1 | F: ACGACTCACTATAGGGCGAA | 539 | 55 | 68K22 |
|  | R: TTTGCGCCGGAATCCACC |  |  |  |
| 68K22-S1 | F: CAGCCAGGGGTTTTCCAGTCAC | 367 | 62 | 427K6 |
|  | R: CGCCGAGCTCCCAAGAACCAG |  |  |  |

* A total of 45 pairs of primers are listed in this table.
