# Supplementary figures and images for: A high-density BAC physical map covering the entire MHC region of addax antelope genome
Source: BMC Genomics. 2019 Jun 11;20:479. doi: 10.1186/s12864-019-5790-2 (PMC6558854; doi:10.1186/s12864-019-5790-2)

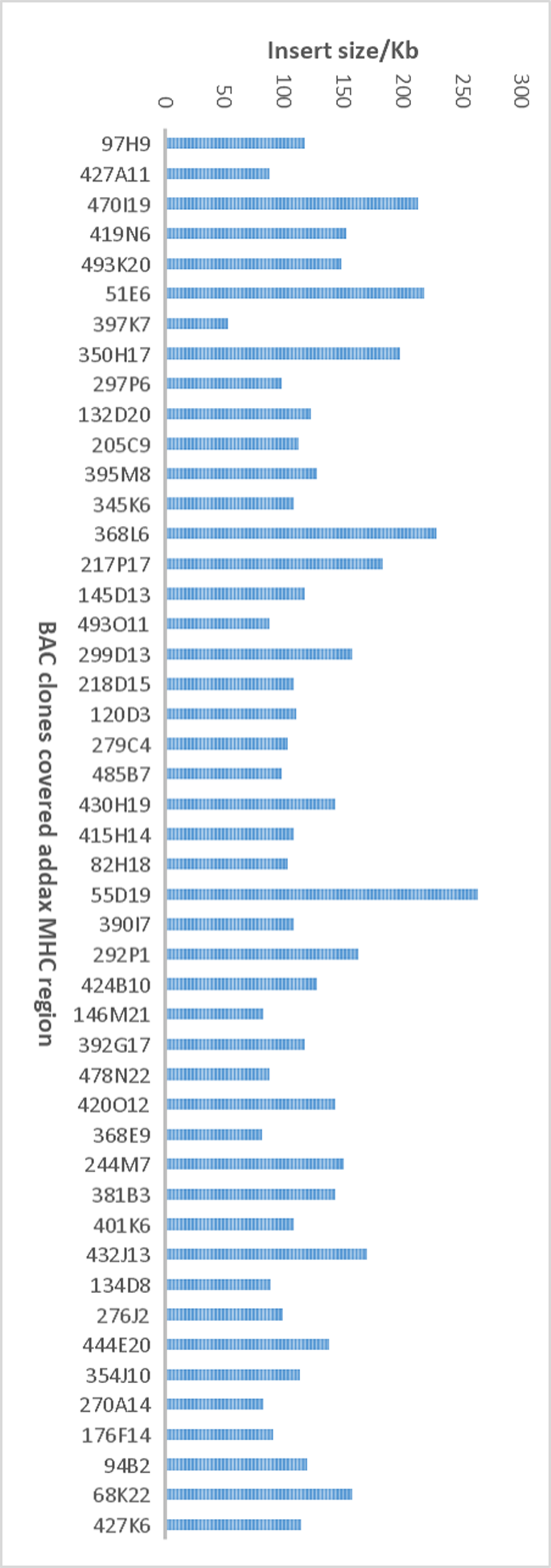


**Fig. S1** **Distribution of insert sizes in the BAC clones covering the addax MHC region**

Supplement: Supplementary file 2 — Figure S1. Distribution of insert sizes in the BAC clones covering the addax MHC region. (DOCX 751 kb) [file 12864_2019_5790_MOESM2_ESM.docx]
