# Supplementary material for: A high-density BAC physical map covering the entire MHC region of addax antelope genome
Source: BMC Genomics. 2019 Jun 11;20:479. doi: 10.1186/s12864-019-5790-2 (PMC6558854; doi:10.1186/s12864-019-5790-2)
Supplement: Supplementary file 3 — Figure S2. Schematic (not to scale) comparing genomic organization of the MHC region in cattle, sheep and addax. The blue, red and green bars represent genes in the MHC class I, III, and II regions, respectively. The bovine and ovine gene maps were adapted from Ensembl and NCBI annotations (not all annotated genes are shown here). (DOCX 363 kb) [file 12864_2019_5790_MOESM3_ESM.docx]

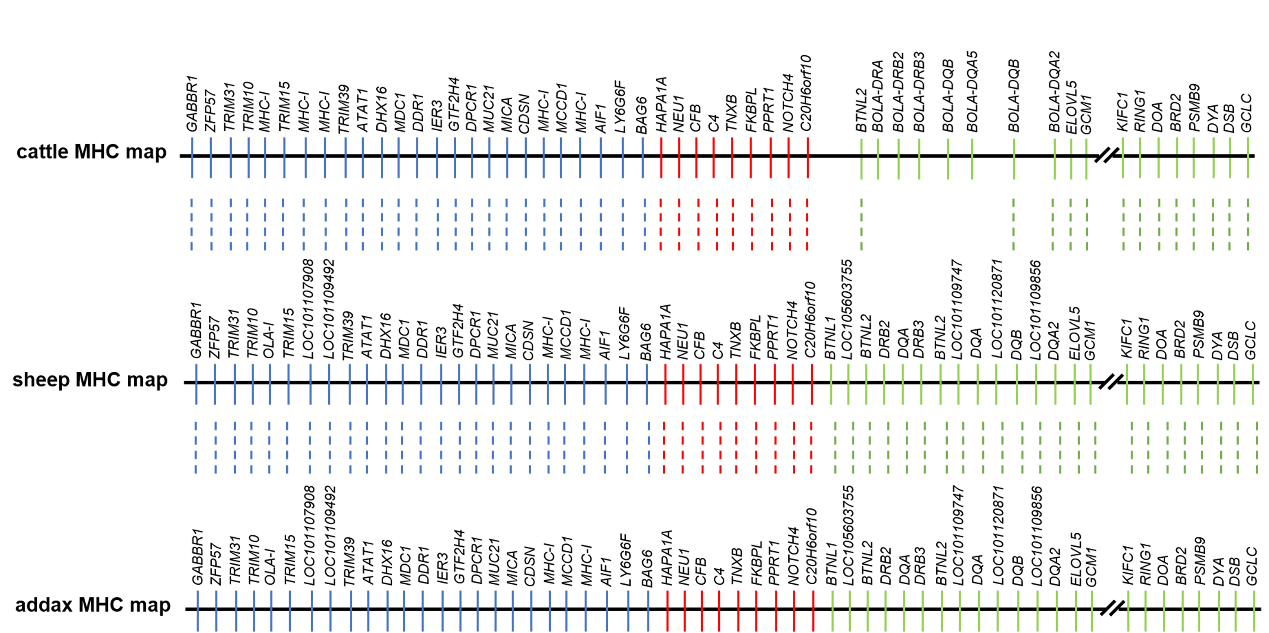


**Fig. S2 Schematic (not to scale) comparing genomic organization of the MHC region in cattle, sheep and addax.** The blue, red and green bars represent genes in the MHC class I, III, and II regions, respectively. The bovine and ovine gene maps were adapted from Ensembl and NCBI annotations (not all annotated genes are shown here).
